# Maternal weight affects placental DNA methylation of genes involved in metabolic pathways in the common marmoset monkey (*Callithrix jacchus*)

**DOI:** 10.1002/ajp.23101

**Published:** 2020-02-05

**Authors:** Laren Narapareddy, Derek E. Wildman, Don L. Armstrong, Amy Weckle, Aleeca F. Bell, Crystal L. Patil, Suzette D. Tardif, Corinna N. Ross, Julienne N. Rutherford

**Affiliations:** ^1^ Nell Hodgson Woodruff School of Nursing Emory University Atlanta Georgia; ^2^ Genomics Program, College of Public Health University of South Florida Tampa Florida; ^3^ Carl R. Woese Institute for Genomic Biology University of Illinois at Urbana‐Champaign Urbana Illinois; ^4^ Illinois Water Resources Center University of Illinois at Urbana‐Champaign Urbana Illinois; ^5^ Department of Women, Children and Family Health Science, College of Nursing University of Illinois at Chicago Chicago Illinois; ^6^ Texas Biomedical Research Institute Southwest National Primate Research Center San Antonio Texas; ^7^ Program of Biology, College of Arts and Sciences Texas A&M University‐San Antonio San Antonio Texas

**Keywords:** developmental programming, epigenetics, maternal metabolism, non‐human primate, obesity

## Abstract

Accumulating evidence suggests that dysregulation of placental DNA methylation (DNAm) is a mechanism linking maternal weight during pregnancy to metabolic programming outcomes. The common marmoset, *Callithrix jaccus*, is a platyrrhine primate species that has provided much insight into studies of the primate placenta, maternal condition, and metabolic programming, yet the relationships between maternal weight and placental DNAm are unknown. Here, we report genome‐wide DNAm from term marmoset placentas using reduced representation bisulfite sequencing. We identified 74 genes whose DNAm pattern is associated with maternal weight during gestation. These genes are predominantly involved in energy metabolism and homeostasis, including the *regulation of glycolytic and lipid metabolic processes pathways*.

## Research Highlights

- The placental DNA methylation (DNAm) landscape of the marmoset placenta presents unique differences and similarities with human placental methylation patterns.
- Maternal weight is associated with placental DNAm in genes that are predominantly involved in energy metabolism and homeostasis.
- The impact of altered placental DNAm on placental function and development may also contribute to the potential role of placental DNAm in developmental programming in the marmoset monkey.

## Graphical abstract

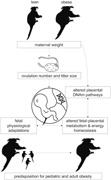

## 1 INTRODUCTION

As part of the gestational interface, the placenta functions through metabolic and endocrine actions that enable a mother to maintain pregnancy and mediates nutrient uptake, waste elimination, and gas exchange for the fetus. As a major regulator of fetal development in utero, the placenta is responsive to both maternal and fetal needs as well as limits. In addition to the immediate purpose that the placenta serves in utero, we now understand that the placenta plays a role in the developmental origins of health and disease. Variation in maternal health (e.g., weight, nutrition, gestational diabetes, psychosocial stress, etc.) alters placental function and regulation of fetal development in ways that affect offspring health outcomes later in life (e.g., obesity, diabetes, hypertension, and pregnancy loss; Burton, Barker, Moffett, & Thornburg, 2011; Burton & Fowden, 2012; Sferruzzi‐Perri & Camm, 2016). Understanding how the placenta orchestrates the interrelationship between mother and fetus during pregnancy is not only important for optimizing birth outcomes, but also for optimizing the long‐term health trajectories of offspring.

Epigenetic regulation is a significant mechanism influencing placental development, structure, and function (Longtine & Nelson, 2011; Nelissen, van Montfoort, Dumoulin, & Evers, 2011; Tarrade, Panchenko, Junien, & Gabory, 2015). Epigenetic modifications occur in all cells and tissues to maintain tissue‐specific development and function. One of the most commonly studied epigenetic modifications is DNA methylation (DNAm; Novakovic & Saffery, 2012). DNAm predominantly occurs at CpG dinucleotide pairs and region of the genome that are cytosine‐/guanine‐rich are known as CpG islands. The DNAm status of CpG sites within gene promoters can affect gene activity (i.e., messenger RNA transcription). Loosely, methylated sites within the promoter allow for repression of gene expression, whereas unmethylated sites within the promoter allow for enhanced gene expression. DNAm is tissue‐ and cell‐type‐specific and thus, evaluating the placenta yields placenta‐specific DNAm patterns (Houseman, Kelsey, Wiencke, & Marsit, 2015). In humans and other species, the low levels of DNAm seen genome‐wide have led to our understanding of the placenta as a globally hypomethylated organ (Schroeder et al., 2015), thus allowing a large proportion of placental genes to be actively transcribed (i.e., expressed). Placental DNAm affects gene expression patterns that play a pivotal role in proper fetal and placental development. The DNAm pattern in the placenta can be disrupted by maternal factors such as nutrition, disease, stress, environmental exposures, and lifestyle factors. For example, human placentas from obese mothers have globally higher levels of DNAm than placentas from nonobese mothers (Nomura et al., 2014). Additionally, maternal glucose concentrations are associated with alterations in placental DNAm of *LEP* (Bouchard et al., 2010; Lesseur et al., 2013; Lesseur, Armstrong, Paquette, et al., 2014), *ADIPOQ* (Bouchard et al., 2012), and *ABCA1* (Houde et al., 2013) genes involved in cellular metabolic processes. An unbiased genome‐wide approach to understanding the associations between the placental DNAm landscape and a range of maternal weights will help identify entire gene pathways whose DNAm is sensitive to maternal weight and the associated maternal milieu.

The common marmoset monkey (*Callithrix jacchus*) is an ideal non‐human primate candidate in which to explore placental DNAm in the context of maternal weight. Our group has demonstrated that the litter size that a mother carries is related to her weight at the time of ovulation (Tardif & Jaquish, 1997). Compared to lighter mothers, heavier mothers ovulate a higher number of ova per cycle and thus, heavier marmoset mothers carry larger litters. An important consequence of litter size is the effect on postnatal growth and long‐term health outcomes (Riesche et al., 2018; Rutherford, deMartelly, Layne Colon, Ross, & Tardif, 2014; Tardif & Bales, 2004; Tardif, Power, Ross, & Rutherford, 2013). Extensive characterization of marmoset placental phenotypes by Rutherford and colleagues (Rutherford, 2007, 2009, 2012; Rutherford & Tardif, 2008, 2009; Rutherford, Eklund, & Tardif, 2009) has demonstrated that compared to twins, triplet placentas demonstrate higher placental efficiency and relative reductions in microscopic surface area (reviewed in Riesche et al., 2018; Rutherford, 2012). Further, the common marmoset was the first neotropical monkey to have its genome sequenced (Worley et al., 2014). This confluence of methods and information can now help us further articulate the role of placental function in mediating maternal ecology and offspring outcomes, both perinatally and into adulthood in the marmoset.

Here, we present for the first time the genome‐wide DNA methylation landscape of the marmoset placenta. Because so much that occurs in downstream development—from number of ova per cycle to litter size born to long‐term developmental and health disparities—is set into motion by maternal weight, we have identified placental genes and gene pathways whose DNAm status is associated with maternal weight during pregnancy, exploring in an important non‐human primate model the hypothesis that maternal weight will be associated with variability in the placental methylome.

## 2 METHODS

### 2.1 Subjects and setting

This study was conducted using animals from two breeding colonies: The Southwest National Primate Research Center (SNPRC) in San Antonio, TX, and the Barshop Institute for Longevity and Aging Studies at the University of Texas Health Science Center at San Antonio. Data and placental tissue were collected from 10 adult female marmoset monkeys that were enrolled in an ongoing project assessing longitudinal and intergenerational effects of maternal development on reproductive outcomes. Between 2012 and 2015, each marmoset female reproduced one to two times per year resulting in data and placental tissue available from a total of 17 pregnancies, produced by a total of 10 dams. Prior, during, and following pregnancy, dams were fed a commercial marmoset diet in ad libitum quantities. All animal procedures, husbandry, and housing comply with the American Society of Primatologists Principles for the Ethical Treatment of Non‐Human Primates and were reviewed and approved by Institutional Animal Care and Use Committees at the University of Illinois at Chicago, Texas Biomedical Research Foundation, and the University of Texas Health Science Center San Antonio.

### 2.2 Maternal weight

Upon detection of pregnancy via ultrasound, estimated gestational age was determined using previously established growth curves (Tardif, Jaquish, Toal, Layne, & Power, 1998). Bodyweight was collected from the female marmosets in their home cage within 1 week of estimated gestational days 60, 90, and 120 (term, ~143 days).

### 2.3 Infant birth outcomes

Birth outcome data was collected as described in Tardif, Layne, Cancino, and Smucny (2002). Briefly, at birth, litter size and sex of the littermates were recorded. Birth weights of each littermate were taken within 24 hr of birth. The sum of individual littermate weights was used as the total litter weight. The average infant weight was calculated as the total litter weight divided by the litter size.

### 2.4 Placental collection and processing

Delivery was not induced in this sample and gestational age and delivery dates were estimated based on growth curves as done in previous work (Tardif et al., 1998). This combined with nocturnal delivery makes the opportunistic placental collection a challenge. However, due to the stable nature of DNAm, both fresh (<1 hr after delivery) and found (1–12 hr after delivery) placentas were included in the study if the placenta was completely intact. Because previous work suggests that the two discs of the marmoset bidiscoid placenta are on the average equivalent in weight and microscopic structure, representing a fully integrated whole (Rutherford & Tardif, 2008), samples for DNAm analysis were collected from a single disc, preserving the second disc for separate analyses. Using a 6‐mm biopsy punch, one central and one peripheral tissue sample were taken yielding two samples per placenta (*N* = 17 placentas, *N* = 34 samples). The tissue samples were immediately placed in RNase‐free tubes with 5 ml of RNAlater (Life Technologies, Austin, TX) and stored at −80° C until further processing.

### 2.5 Placental DNA purification and quantification

Placental samples weighing from 7 to 25 mg were obtained for total DNA purification using the DNeasy Blood & Tissue Kit (Qiagen, Valencia, CA). All reagents and equipment in the manufacturer's protocol were followed with the addition of RNase A digestion to yield RNA‐free genomic DNA. Genomic DNA was quantified using two dsDNA assay methods: Quant‐iT Picogreen (Invitrogen, Carlsbad, CA) and Qubit System (Thermo Fischer Scientific, Waltham, MA). The quality of the DNA was further assessed through gel electrophoresis.

### 2.6 DNA methylation libraries, sequencing, and alignment

Reduced representation bisulfite sequencing (RRBS) was performed on 100 ng of genomic DNA input at the Roy J. Carver Biotechnology Center at the University of Illinois in Urbana, IL, using Ovation RRBS Methyl‐Seq System 1–16 (NuGEN Technologies, San Carlos, CA). The 34 samples were randomly assigned to five lanes and the samples were run in five pools, four lanes with seven samples and one lane with six samples. RRBS uses *Msp*I which is a methylation insensitive restriction enzyme that recognizes CCGG sites. As *Msp*I is insensitive to methylation status, all CCGG sites were cut between the two C's resulting in small fragments with a high frequency of potential CpG methylation sites. Following *Msp*I digestion, the fragments underwent adaptor ligation, final repair, bisulfite conversion, and polymerase chain reaction (PCR) amplification to produce the final libraries for sequencing. The five pools were sequenced on five lanes in the HiSeq 4000 (Illumina, San Diego, CA) with paired‐end reads 100 nt in length. Trimming was performed using Python scripts provided by NuGen to remove low‐quality bases, adaptor sequences, and the sequence diversity provided by the NuGEN RRBS adaptor. The trimmed bisulfite sequence was then aligned to the marmoset genome (C_jacchus 3.2.1, version 85) using the bwa‐meth package in R which uses bwa‐mem to perform the alignment (Pedersen, Eyring, De, Yang, & Schwartz, 2014). PCR duplication artifacts were removed, and methylation status was determined using the PileOMeth package in R which extracts per‐base methylation metrics. Data have been deposited at Gene Expression Omnibus (GEO; GSE143200).

### 2.7 Statistical analyses

All analyses were conducted using R (version 3.3.2) and RStudio (version 1.0.136) with packages available through CRAN and Bioconductor. Each placenta was treated independently given that previous work by our group has demonstrated high within‐subject variability in litter size and placental phenotypic characteristics from pregnancy to pregnancy (Rutherford & Tardif, 2008; Tardif & Jaquish, 1997).

**Figure 1:**
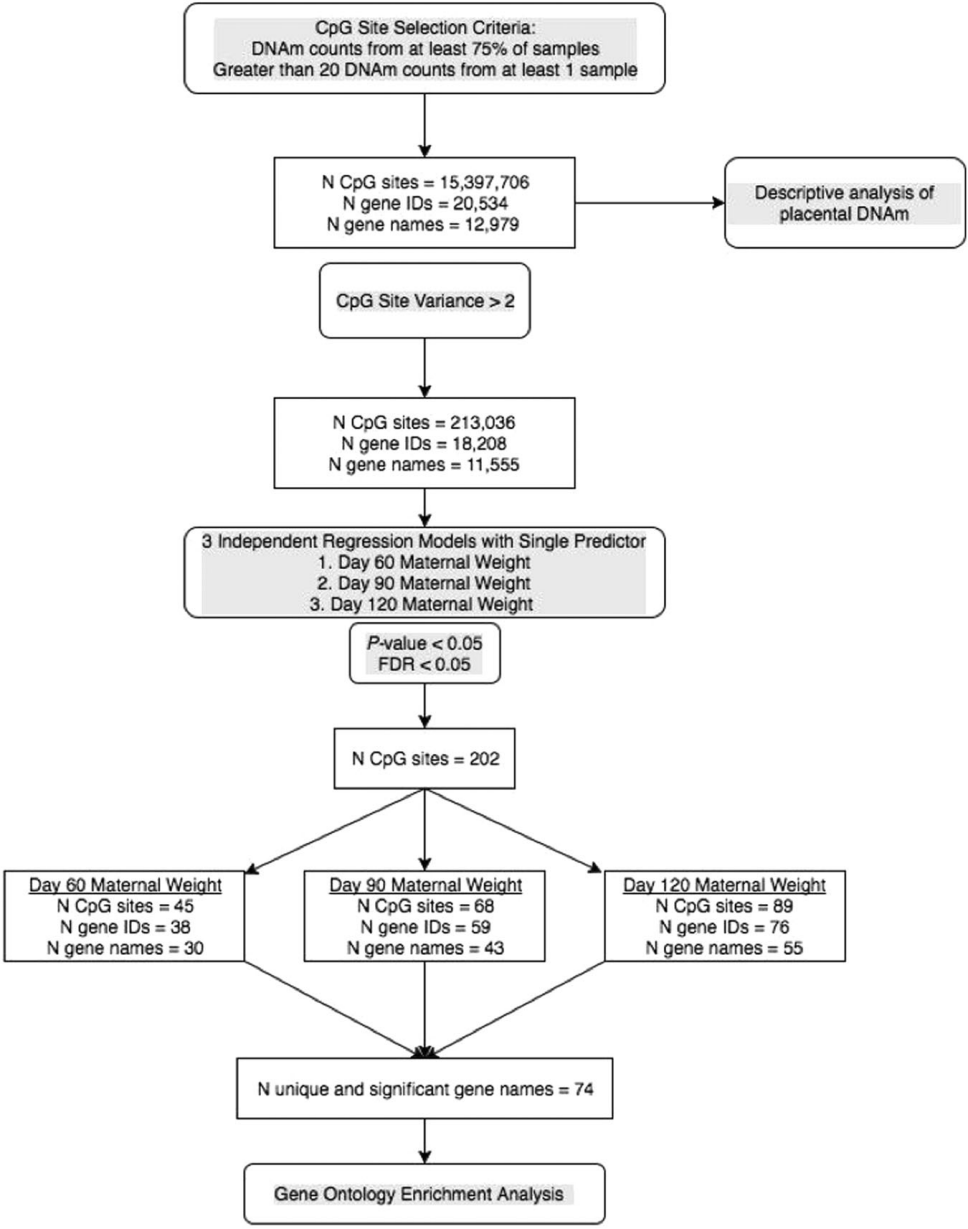
Workflow of the analytical strategy used to describe DNA methylation (DNAm) in the marmoset placenta and to identify genes and gene pathways that are associated with maternal weight during gestation

**Figure 2:**
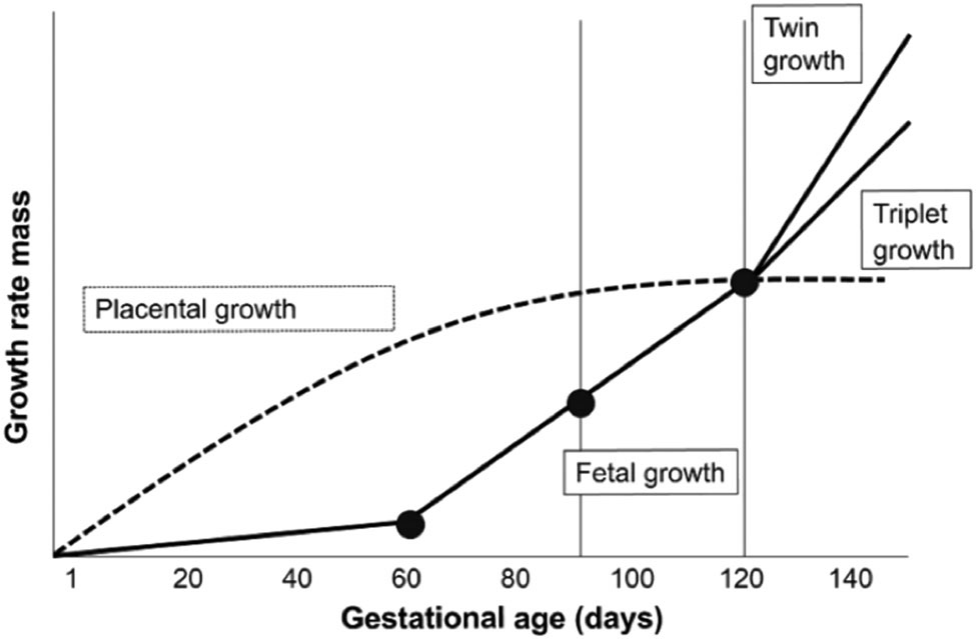
Schematic timeline of marmoset fetoplacental development across 143 days of gestation, indicating critical time points (•) of the model. The most rapid period of placental growth occurs between Days 0 and 100, at which time placental growth is completed. Fetal growth is slow during early gestation until Day 60 when it begins to rapidly increase through Day 120. Day 90 is the midway point of this growth rate period. The most rapid increase in fetal growth rate occurs after Day 120 and at this time point, twin growth rate exceeds triplet growth rate. From Riesche et al. (2018; permissions will be requested)

Although methylation can occur and be detected at non‐CpG sites (i.e., CpHpG and CpHpH) through RRBS, only CpG sites were evaluated in this study. For inclusion in analyses, we required CpG sites to have the counts from at least 75% of the samples with at least one sample to have greater than 20 counts. Using the methylation count data, *β*‐values and *M*‐values at each CpG site were calculated using the following equations: *β* = methylated counts/(methylated counts + unmethylated counts); *M* = *β*/(1−*β*) (Du et al., 2010). For statistical analyses, the *M*‐values were used to promote normality and reduce heteroscedasticity of the data and allow for better alignment with statistical assumptions (Du et al., 2010; Wright et al., 2016). Correlations were assessed using Pearson's pairwise correlation tests. Figure 1 shows the analysis workflow. In CpG sites with variance >2, the association between maternal weight and placental DNAm was assessed by three separate regression models with single predictors: maternal weight at (a) gestational Day 60; (b) gestational Day 90; and (c) gestational Day 120. We selected gestational Day 60, when the fetal growth rate drastically increases; Day 90, when placental growth rate begins to plateau; and Day 120, when triplet growth rate begins to decrease in comparison to growth rate of twins (Figure 2). Following regression, a false discovery rate (FDR) < 0.05 was applied using the Benjamini–Hochberg method (Benjamini & Hochberg, 1995) and CpG sites that were significantly related to maternal weight at any time point were used to create a predefined gene list for gene ontology analysis. A semi‐automated enrichment analysis for gene ontology categories was performed using the topGO package in R (Alexa & Rahnenführer, n.d.). A *P* value based on Fisher's exact test was provided to identify gene ontology categories that are enriched (i.e., over‐represented in the predefined list of genes). It is important to note that enrichment does not mean an overall category or its constitutive genes are hypermethylated or hypomethylated; it does not imply directionality relative to maternal weight category. Adjustment for multiple comparisons was done using the Benjamini–Hochberg method with an FDR < 0.05.

## 3 RESULTS

### 3.1 Sample descriptive statistics

Maternal, placental, and infant characteristics are presented in Table 1. Seventeen pregnancies resulted in litters ranging in size from two to five, with triplets being the most frequent (*n* = 8). Total litter weight increased with respect to litter size, and average infant weight for the sample was 29.64 g. Maternal weight was assessed at three different time points throughout gestation: (a) gestational Day 60 (42% of term); (b) gestational Day 90 (63% of term); and (c) gestational Day 120 (84% of term). The sample demonstrated a wide range in maternal weight at each time point, however, as expected, mean maternal weight increased throughout gestation. Quintuplet pregnancies are a rare occurrence and when the one quintuplet pregnancy that occurred in this sample was removed from analyses, maternal weight was significantly correlated with litter weight at each gestational time point (*n* = 16; *r* = 0.44 at 60 days, *r* = .58 at 90 days, *r* = .64 at 120 days; *p* < .05), as well as with litter size at gestational Day 120 (*r* = .39, *p* < .05).

**Table 1:** Maternal, placental, and infant characteristics

	*N*	Mean ± standard deviation	Range
Maternal weight (g)			
60‐day weight	17	440.47 ± 89.09	318.2–614.0
90‐day weight	17	464.56 ± 93.93	360.2–656.0
120‐day weight	17	507.61 ± 88.90	412.0–702.0
Placental outcomes			
Placental weight (g)	17	8.66 ± 2.56	6.2–14.9
Placental volume (ml)	17	8.82 ± 3.38	5.0–17.0
Placental efficiency	17	9.93 ± 3.39	6.48–19.85
Infant outcomes (g)			
Total litter weight	17	83.04 ± 26.81	47.4–134.4
Average infant weight	17	29.64 ± 6.25	15.8–44.8
Infant weight (g) by litter size			
Twin	4	32.02 ± 2.15	28.95–34.00
Triplet	8	26.69 ± 5.93	15. 80–36.13
Quadruplet	4	29.41 ± 2.59	26.59–32.50
Quintuplet	1	44.80	–

### 3.2 Placental DNA methylation analyses

**Figure 3:**
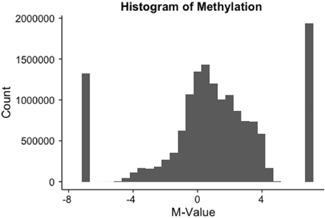
Distribution of methylation across all CpG sites. Histogram of *M*‐Value. *M* = −7 indicated fully unmethylated CpG sites, *M* = 0 indicates hemi‐methylated CpG sites, and M = 7 indicates fully methylated CpG sites

After applying our inclusion criteria to RRBS data from 17 placental samples, a total of 15,397,706 CpG sites were included. This corresponds to 18,208 marmoset gene IDs and 11,555 unique gene names. Table S1 summarizes the sequencing reads obtained per CpG site and per sample. The distribution of DNAm across all CpG sites is shown in Figure 3. An *M*‐value of −7 represents completely unmethylated CpG sites, an *M*‐value of 7 represents completely methylated CpG sites, and an *M*‐value of zero represents hemimethylated CpG sites (i.e., CpG sites with an equal number of sequencing reads that were methylated and unmethylated). The sequencing read data and distribution of DNAm demonstrate a slight negative skew with a higher proportion demonstrating *M*‐values greater than zero. Sixty‐five percent of CpG sites had an *M*‐value greater than zero with 12% of CpG sites demonstrating complete methylation.

DNAm within gene regions was calculated using the reads that were aligned within the ±3‐kb region of the transcription start site (TSS; i.e., the promoter region). The general pattern observed was that DNAm decreased in the upstream region as it approached the TSS. DNAm was lowest at the TSS and increased as the downstream distance from the TSS increased. In this study, 0.06% of CpG sites captured were located at a TSS and 32.97% were within the promoter region.

**Figure 4:**
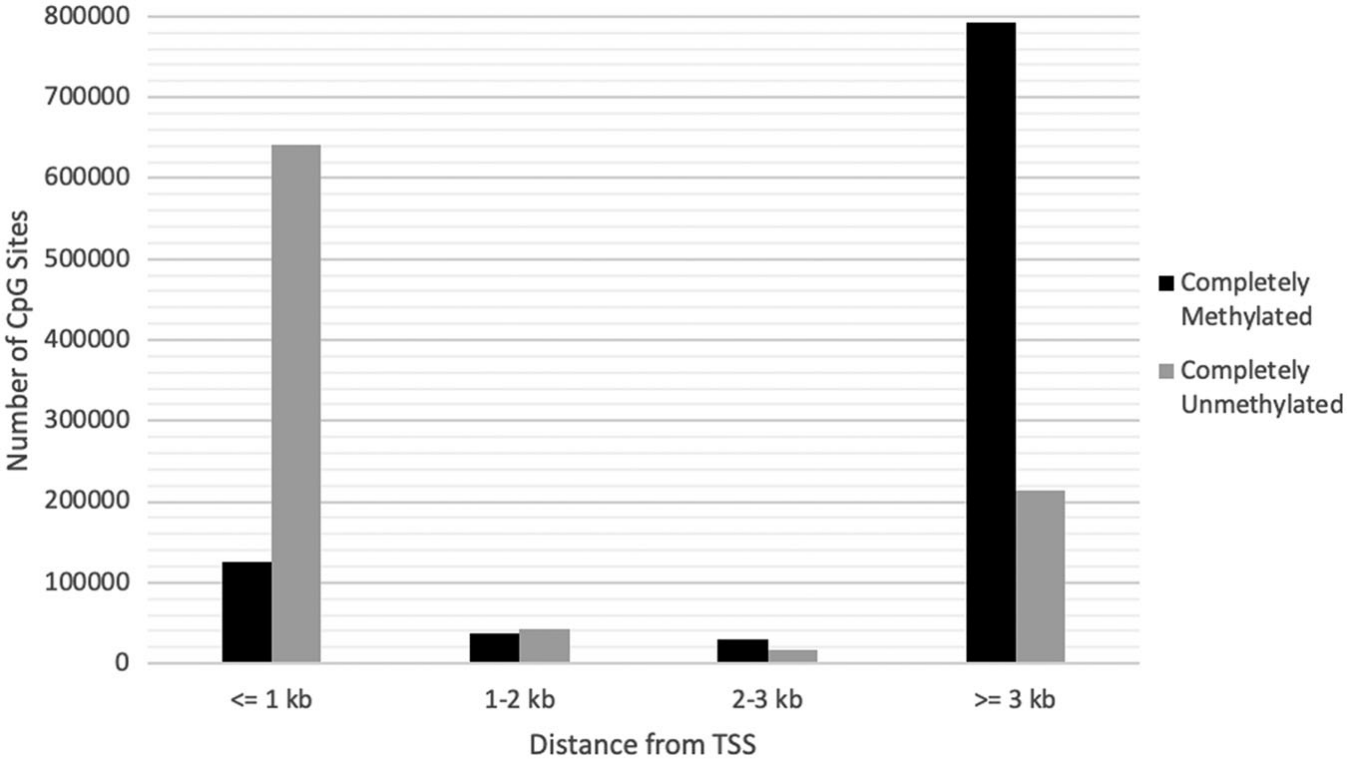
Comparison of the number of completely methylated (▪) and completely unmethylated (▪) CpG sites at specific distances within (<3 kb) and outside (≥3 kb) the promoter region

Completely methylated and completely unmethylated CpGs were compared within (<3 kb) and outside (≥3 kb) the promoter region to explore any patterns in the occurrence of completely methylated and completely unmethylated CpG sites in the marmoset placenta. Figure 4 demonstrates a high occurrence of completely unmethylated CpGs within the promoter region and a high occurrence of completely methylated CpGs outside the promoter region.

### 3.3 Placental DNAm and maternal weight

The number of significant CpG sites (*p* < .05, FDR 0.05), unique gene identifiers, and unique gene names for each maternal weight predictor are shown in Figure 1 and Tables S2–S4 provide lists of gene names and further details that are unique for each predictor. The unique gene names that were significantly associated with DNAm for each predictor were combined and de‐duplicated to create a predefined gene list for gene ontology analysis which contained 74 genes that were compared to the reference gene list of 11,555 genes. Fisher's exact test was used for comparison. These 74 genes are listed in Table S5 along with the functional and biological annotation in the marmoset as obtained through Database for Annotation, Visualization, and Integrated Discovery (Huang, Sherman, & Lempicki, 2009a, 2009b). Given the number of comparisons that must be controlled for in genome‐wide analyses, it is not surprising that after applying an FDR < 0.05, none of the gene ontology categories remained significant. Table 2 lists the top 20 gene ontology categories that were enriched in relation to maternal weight. The top four categories were *regulation of glycolytic processes* (GO:0006110, 26 annotated genes), *carnitine shuttle* (GO:0006853, 6 annotated genes), *regulation of carbohydrate catabolic processes* (GO:0043470, 31 annotated genes), and *regulation of lipid metabolic processes* (GO:0019216, 188 annotated genes).

**Table 2:** Top 20 gene ontology terms that demonstrate differential methylation based on maternal weight

GO ID	GO term	Annotated genes	*P* value	FDR
GO:0006110	Regulation of glycolytic process	26	.00047	1
GO:0006853	Carnitine shuttle	6	.00051	1
GO:0043470	Regulation of carbohydrate catabolic process…	31	.00079	1
GO:0019216	Regulation of lipid metabolic process	188	.00082	1
GO:0009118	Regulation of nucleoside metabolic process…	37	.00133	1
GO:1903578	Regulation of ATP metabolic process	37	.00133	1
GO:0051193	Regulation of cofactor metabolic process	43	.00206	1
GO:0051196	Regulation of coenzyme metabolic process	43	.00206	1
GO:0006096	Glycolytic process	46	.00251	1
GO:0006757	ATP generation from ADP	46	.00251	1
GO:0042451	Purine nucleoside biosynthetic process	47	.00267	1
GO:0046129	Purine ribonucleoside biosynthetic process…	47	.00267	1
GO:0046031	ADP metabolic process	51	.00337	1
GO:0010882	Regulation of cardiac muscle contraction…	15	.00345	1
GO:0046128	Purine ribonucleoside metabolic process	180	.00416	1
GO:0030258	Lipid modification	181	.00426	1
GO:0042278	Purine nucleoside metabolic process	182	.00436	1
GO:0006165	Nucleoside diphosphate phosphorylation	56	.00439	1
GO:0046034	ATP metabolic process	114	.00458	1
GO:0046939	Nucleotide phosphorylation	58	.00484	1

Abbreviation: FDR, false discovery rate

## 4 DISCUSSION

### 4.1 The unique DNA methylation landscape in the marmoset monkey placenta

In humans and other mammals, the placenta is a globally hypomethylated organ (reviewed in Schroeder et al., 2015) with recent RRBS reports of 36–39% DNAm in human third‐trimester placenta (Chatterjee et al., 2016). In contrast, the marmoset placenta exhibits a higher than expected level of methylation, with 65% of the covered CpG sites demonstrating *M*‐values greater than zero, suggesting that marmoset placentas may be more globally methylated at CpG sites than human placentas.

While the present study captured a higher extent of genome‐wide DNAm than expected, the pattern of methylation in the marmoset placenta may be similar to the human placenta in that hypomethylation is not uniform across the entire placental genome (Robinson & Price, 2015). Typically, in somatic and germline tissues there is a global pattern of complete methylation of CpG sites across the genome, with the exception of CpG sites that lie within the promoter region which demonstrate high variability based on tissue type (Jiang et al., 2014). Within the gene promoters, CpG site methylation usually inhibits transcription by limiting transcription factor binding accessibility which leads to gene silencing (Koukoura, Sifakis, & Spandidos, 2012). Conversely, unmethylated promoters are usually actively transcribed and expressed (Jones, 2012).

Both marmoset and human demonstrate low levels of methylation within gene promoter regions and higher levels of DNAm outside of promoters. Our data suggest that the greater extent of DNAm outside the promoter in marmosets may be due to differences in CpG site density between the two genomes. It has yet to be explored whether or not the higher levels of DNAm outside the gene promoters contribute to functional changes that are specific to marmoset placentas. Yet, it is likely that the lower levels of methylation that occur within gene promoter regions of the marmoset placenta lead to similar patterns of increased transcription and expression that are characteristic of the mammalian placenta (Novakovic et al., 2011). Though the absolute amount of DNA methylation appears to differ between humans and marmosets, the pattern and function of DNA methylation may be quite similar.

### 4.2 Maternal weight is associated with placental DNAm in genes that are predominantly involved in energy metabolism and homeostasis

An important and biologically significant aspect of our results in the marmoset is that heavier marmosets exhibit metabolic alterations such as increased fasting glucose, hemoglobin A1C, triglycerides, and very low‐density lipoproteins (Tardif et al., 2009). Although our analysis do not use specific maternal weight categories to determine which individual genes are hyper‐ or hypomethylated, it does suggest that the maternal metabolic milieu influences placental DNAm in genes involved in key metabolic pathways such as glycolysis, carbohydrate catabolism, and lipid metabolism. Such changes in DNA methylation of these pathways could serve to increase the overall availability of nutrients to the developing fetoplacental unit, potentially allowing larger mothers to gestate a greater number of fetuses.

The placenta is a metabolically active tissue that conducts significant nutrient uptake from the maternal circulation. For example, in the human placenta, 50% of the glucose taken up from maternal circulation is metabolized via glycolysis to serve the metabolic needs of the placenta (Illsley, 2011). The maternally sourced nutrients are metabolized by the placenta and used for generating cellular energy and synthesizing components essential for cellular growth within the placenta such as DNA, RNA, proteins, membranes, and other biological building blocks (Illsley, 2011). Though the consumption and disposition of glucose and other nutrients in the marmoset placenta are currently unknown, genes that are a part of the *regulation of glycolytic processes* and *regulation of carbohydrate catabolic processes* pathway will be of particular interest in future studies given that glucose is the primary substrate that the placenta uses to generate energy for growth and development of the fetoplacental unit. Future studies exploring placental DNAm in a multilevel context inclusive of gene and protein expression as well as nutrient consumption and disposition will contribute to a better understanding of how the maternal metabolic milieu influences the metabolic dynamics of the marmoset placenta through placental DNAm of genes within the *regulation of glycolytic processes* and *regulation of carbohydrate catabolic processes* pathways.

### 4.3 The potential role of placental DNAm in developmental programming in the marmoset

We've previously reported on the developmental programming outcomes and placental phenotypic differences that are observed in the marmoset based on litter size (reviewed in Riesche et al., 2018). Central to the common marmoset monkey as a model of developmental programming is that heavier mothers carry larger litters with immediate‐, short‐, and long‐term health disparities. Triplets are more likely to grow from low weights at birth to high weights in adulthood (“centile crossing”) and compared to twins, they are more likely to develop obesity (Tardif & Bales, 2004) and decreased reproductive potential (Rutherford et al., 2014) later in life. Though mothers of triplets are heavier at the outset of pregnancy, their gestational weight gain is not proportional to that of mothers carrying twins; there is a 24.9% average decrease in maternal‐to‐fetal mass ratio in triplets compared to twins (Rutherford & Tardif, 2008). Thus, the additional fetuses gestated by larger mothers create an intrauterine environment of relatively higher fetal demand and lower maternal energetic resources (Rutherford & Tardif, 2008). The placental phenotypic differences between twins and triplets (including decreased surface area of the maternal‐fetal interface per fetus and decreased IGF‐II concentrations per fetus (Rutherford et al., 2009; Rutherford & Tardif, 2009) suggest that the placenta dynamically alters its structure and function in relation to altered fetal demands and maternal supply in a way that may influence fetal development and long‐term health outcomes.

**Figure 5:**
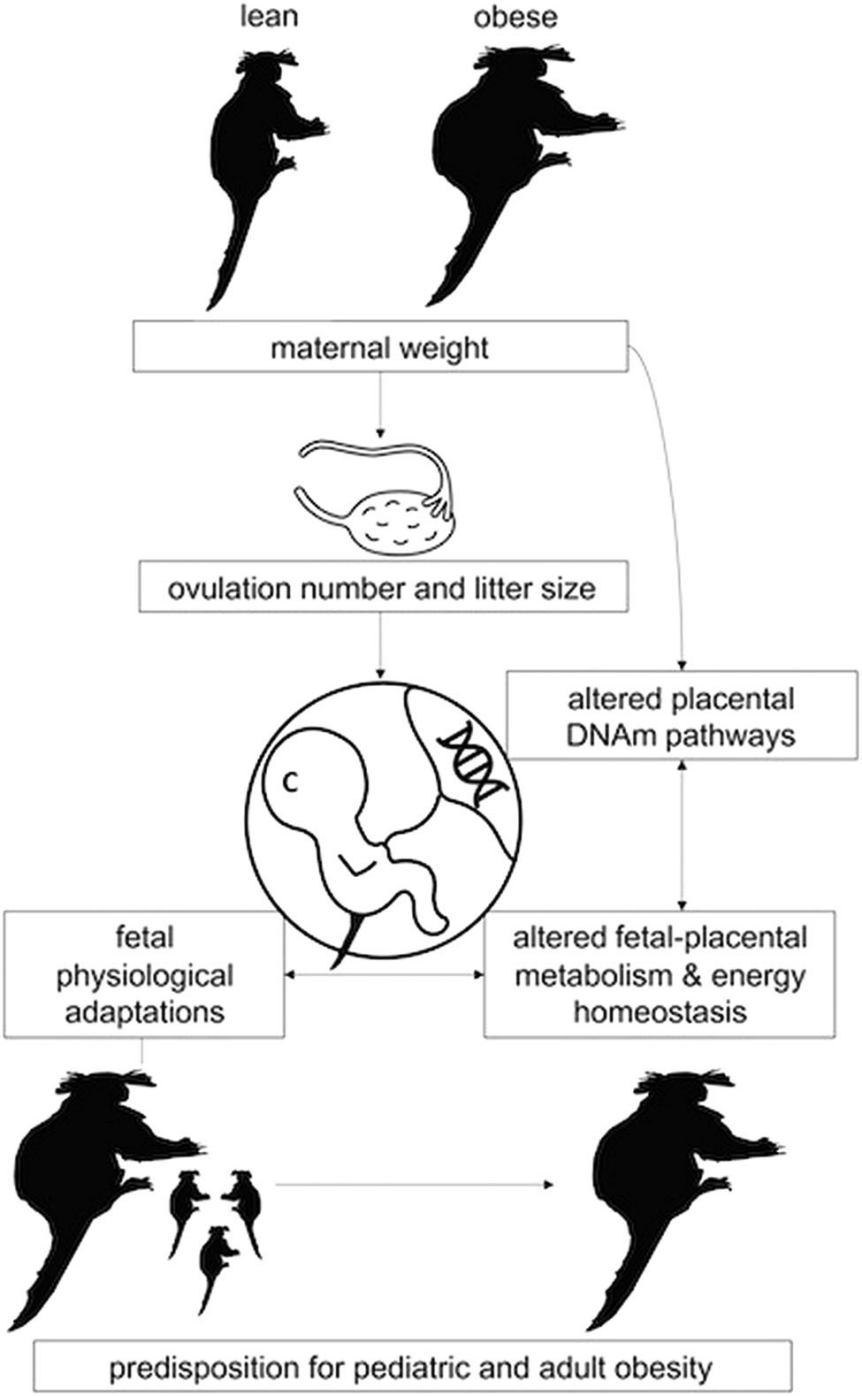
Current model demonstrating the potential role of placental DNA methylation (DNAm) in developmental programming of obesity. Maternal weight influences ovulation number and is related to placental DNAm patterns in gene pathways involved in energy metabolism and homeostasis. Downstream effects of altered DNAm in these pathways likely leads to physiological adaptations in the fetus with consequences for long‐term metabolic health

Our results here demonstrate that one way in which the placenta may negotiate higher fetal demand and lower relative maternal energetic supply in larger marmoset mothers is through placental DNA methylation in genes and gene pathways involved in supplying glucose and lipids to the fetus. The consequences of alterations in these pathways may influence fetal growth and development in ways that impact the health trajectories of offspring born to larger litters and are likely involved in the health disparities observed between offspring from different litter sizes (Figure 5). Recent work in maternal obesity provides evidence for altered placental metabolic and energetic processes that are within the pathways that we have identified here. These processes include changes in fatty acid and lipid transport, decreased mitochondrial energetics, decreased ATP generation in the trophoblast, and oxidative stress in the placentas of obese pregnancies (reviewed in Myatt & Maloyan, 2016). Formation of placental lipid pools may be one mechanism that modulates fatty acid transfer to the fetus (Perazzolo et al., 2017). An ovine model demonstrated significantly higher free fatty acid levels in the fetal circulation of obese pregnancies which may contribute to programming fetal lipid metabolism (Zhu, Du, Nathanielsz, & Ford, 2010). Whether or not placental DNAm in these pathways has a role in such mechanisms of developmental programming in the marmoset placenta remains to be explored. As the cohort begins to reach adulthood, we are exploring the role of placental epigenetics in developmental programming by determining whether placental epigenetic profiles are related to placental structural phenotypes, birth outcomes, and long‐term health outcomes in offspring.

### 4.4 Limitations and future directions

We assumed that the marmoset genome acts similarly to the human genome in the orchestration and execution of biological processes and that DNAm has the same impacts on gene transcription and expression as is known in other species. Considering the phylogenetic similarities and shared sequence homology among primates it is likely that these similarities do exist, however, further studies will be necessary to rule out evolutionary differences and variants that may impact the dynamics of the marmoset genome and DNAm. For example, marmosets differ from humans in their type of maternal‐placental pattern of interdigitation with trabecular and villous interdigitations, respectively (Mossman, 1987). To that end, our team is in the process of assessing the association between gene expression and DNAm in the marmoset placenta. Cellular heterogeneity and gestational age are additional factors that we were not able to explore in the current study, but it will be important to design future studies to elucidate the influence of these factors. Placental sex plays an important role in offspring outcomes (Clifton, 2010); however placental sex as a biological confounder will be difficult to account for in all studies of the marmoset placenta due to interfetal chimerism (Benirschke, Anderson, & Brownhill, 1962; Ross, French, & Orti, 2007) and the high frequency of mixed‐sex litters (Rothe, Darms, & Koenig, 1992). Finally, we were able to detect enrichment of gene categories across a continuous range of maternal weight. Enrichment includes overrepresentation of both hypermethylated and hypomethylated genes; as of yet, we have not determined the specific weight‐related methylation status of the thousands of genes represented in our analyses. These foundational findings point us in the direction of candidate genes to target for further study relative to maternal, placental, and offspring phenotypes.

As this is the first study to report genome‐wide DNAm in the marmoset monkey, many avenues are now open for further elucidating the epigenetic characteristics and mechanisms of the marmoset placenta. These avenues, which are beyond the scope of this current study, include exploring how directional changes in DNAm relate to gene and protein expression as well as overall functional changes in metabolic and energetic pathways within the placenta. Another exciting avenue which we are situated to begin exploring is what the associations between maternal weight and placental DNAm mean for fetal development and offspring outcomes. As with the current hypothesis that parturition is governed by the balance between maternal and fetal metabolism (Dunsworth, Warrener, Deacon, Ellison, & Pontzer, 2012), it is possible that there is a point at which maternal metabolic effects on placental DNAm status confer adverse metabolic outcomes in the offspring. The findings reported here provide fertile ground for future studies to further elucidate our understanding of maternal obesity, developmental programming, and the marmoset placenta.

## CONFLICT OF INTERESTS

The authors declare that there are no conflict of interests.

### OPEN RESEARCH BADGES

This article has earned an Open Data badge for making publicly available the digitally‐shareable data necessary to reproduce the reported results. The data is available at https://www.ncbi.nlm.nih.gov/geo/query/acc.cgi?acc=GSE143200.

## Supporting information

Supporting information

## Data Availability

The data that support the findings of this study are openly available in GEO (GSE143200).
