# Supplementary material for: Maternal weight affects placental DNA methylation of genes involved in metabolic pathways in the common marmoset monkey (Callithrix jacchus)
Source: Am J Primatol. 2020 Feb 5;82(3):e23101. doi: 10.1002/ajp.23101 (PMC7154656; doi:10.1002/ajp.23101)
Supplement: Supplementary file 1 — Supporting information [file AJP-82-e23101-s001.pdf]

Table S1

Sequencing reads obtained by whole-genome RRBS.

	N (reads)	Mean (reads)	Range (reads)
Per CpG site (N=15,397,706)			
Total reads	229,080,095	14.9	1-484
Methylated reads	141,710,362	9.2	0-433
Unmethylated reads	87,369,733	5.7	0-363
Per sample (N=34)			
Total reads	-	6,737,649.9	6,272,810 - 7,333,074
Methylated reads	-	4,167,951.8	3,941,390 - 4,444,733
Unmethylated reads	-	2,569,698.0	2,303,802 - 2,889,340

Table S2

45 *CpG sites significantly associated with maternal weight at gestational day 60 in descending order of P value with FDR <0.05. Alphanumeric chromosomes represent unassembled contigs.*

Chromosome	Start Position	Gene Name	Gene ID	*P* value	FDR
10	16507933	NA	ENSCJAG00000032743	8.21E-09	0.00075
5	135692805	*5S_rRNA*	ENSCJAG00000024555	2.04E-08	0.00163
9	13850661	*CACNA1C*	ENSCJAG00000000732	6.67E-08	0.00251
1	177324707	NA	ENSCJAG00000017966	1.01E-07	0.00287
1	207684919	NA	ENSCJAG00000018966	1.03E-07	0.00287
4	1639040	NA	ENSCJAG00000022438	1.26E-07	0.00310
1	177313750	NA	ENSCJAG00000017966	2.16E-07	0.00446
3	183761230	*CRMP1*	ENSCJAG00000010648	2.37E-07	0.00470
3	183761811	*CRMP1*	ENSCJAG00000010648	3.69E-07	0.00553
18	2316759	*PRKAB2*	ENSCJAG00000002530	5.04E-07	0.00685
2	3048585	*WBSCR17*	ENSCJAG00000005178	5.70E-07	0.00739
22	12418791	*CACNA1A*	ENSCJAG00000004458	9.00E-07	0.01031
22	21451357	NA	ENSCJAG00000032315	1.03E-06	0.01112
1	195085254	*FBXO7*	ENSCJAG00000006238	1.17E-06	0.01200
6	155933814	NA	ENSCJAG00000034894	1.39E-06	0.01289
22	21451391	NA	ENSCJAG00000032315	1.71E-06	0.01542
1	141811277	*ANKS6*	ENSCJAG00000011142	1.75E-06	0.01557
16	71400669	*WDYHV1*	ENSCJAG00000000865	2.10E-06	0.01742
18	36565866	*SCYL3*	ENSCJAG00000010050	2.35E-06	0.01925
7	36083382	*TMEM240*	ENSCJAG00000037689	2.80E-06	0.02047
7	49305869	*KAZN*	ENSCJAG00000008920	2.82E-06	0.02047
18	2316773	*PRKAB2*	ENSCJAG00000002530	2.90E-06	0.02073

ACFV01190020.1	1340	NA	NA	3.11E-06	0.02162
7	37009749	*TNFRSF14*	ENSCJAG00000002555	3.48E-06	0.02304
1	177313680	NA	ENSCJAG00000017966	3.64E-06	0.02346
16	91386366	*BAALC*	ENSCJAG00000016141	4.31E-06	0.02462
1	177956403	*VAV2*	ENSCJAG00000010024	4.51E-06	0.02504
12	84494722	*PGAM1*	ENSCJAG00000016575	4.76E-06	0.02577
9	88988805	*ANKS1B*	ENSCJAG00000002273	5.60E-06	0.03003
6	157494660	*CROCC2*	ENSCJAG00000006365	5.64E-06	0.03003
20	11687025	*ADGRG1*	ENSCJAG00000011920	6.36E-06	0.03025
5	126698006	*SEPT9*	ENSCJAG00000013203	6.47E-06	0.03025
2	192181103	*CTNND2*	ENSCJAG00000009238	6.55E-06	0.03025
15	5509601	NA	ENSCJAG00000031315	7.06E-06	0.03155
3	67058583	*U6*	ENSCJAG00000027016	7.47E-06	0.03182
GL285864.1	261	NA	NA	8.05E-06	0.03317
2	202460667	*Y_RNA*	ENSCJAG00000028941	8.22E-06	0.03329
3	183762034	*CRMP1*	ENSCJAG00000010648	8.45E-06	0.03384
22	7374258	*TRAPPC5*	ENSCJAG00000036638	8.47E-06	0.03384
2	200569162	*NA*	ENSCJAG00000036280	9.41E-06	0.03577
6	12987155	*MCTP2*	ENSCJAG00000005975	9.94E-06	0.03705
11	109603018	*DGKZ*	ENSCJAG00000011773	1.11E-05	0.03992
4	34401203	*BAK1*	ENSCJAG00000016047	1.15E-05	0.04090
13	10797937	*EPHX2*	ENSCJAG00000019677	1.39E-05	0.04601
GL285558.1	1187	*snoU13*	ENSCJAG00000036742	1.52E-05	0.04799

Table S3

68 *CpG sites significantly associated with maternal weight at gestational day 90 in descending order of P value with FDR <0.05. Alphanumeric chromosomes represent unassembled contigs.*

Chromosome	Start Position	Gene Name	Gene ID	*P* value	FDR
4	1639040	NA	ENSCJAG00000022438	6.00E-10	0.00019
10	16507933	NA	ENSCJAG00000032743	1.88E-09	0.00024
1	207684919	NA	ENSCJAG00000018966	2.75E-08	0.00177
2	3048585	*WBSCR17*	ENSCJAG00000005178	3.04E-08	0.00177
1	177324707	NA	ENSCJAG00000017966	3.69E-08	0.00196
9	13850661	*CACNA1C*	ENSCJAG00000000732	4.12E-08	0.00202
1	177956403	*VAV2*	ENSCJAG00000010024	1.01E-07	0.00287
3	183761230	*CRMP1*	ENSCJAG00000010648	1.03E-07	0.00287
6	155933814	NA	ENSCJAG00000034894	1.20E-07	0.00310
6	155718341	*HDAC4*	ENSCJAG00000009423	1.45E-07	0.00330
1	177313750	NA	ENSCJAG00000017966	1.73E-07	0.00375
7	36083382	*TMEM240*	ENSCJAG00000037689	2.43E-07	0.00470
22	12418791	*CACNA1A*	ENSCJAG00000004458	2.69E-07	0.00479
1	177313680	NA	ENSCJAG00000017966	3.39E-07	0.00548

18	36565866	*SCYL3*	ENSCJAG00000010050	3.58E-07	0.00553
3	186729794	NA	ENSCJAG00000031790	4.93E-07	0.00685
13	10797937	*EPHX2*	ENSCJAG00000019677	5.78E-07	0.00739
5	135692805	*5S_rRNA*	ENSCJAG00000024555	6.00E-07	0.00752
2	192181103	*CTNND2*	ENSCJAG00000009238	9.04E-07	0.01031
6	157494660	*CROCC2*	ENSCJAG00000006365	1.02E-06	0.01112
2	3048584	*WBSCR17*	ENSCJAG00000005178	1.18E-06	0.01200
12	118225906	NA	ENSCJAG00000014400	1.20E-06	0.01200
1	181715957	*EXD3*	ENSCJAG00000013170	1.28E-06	0.01223
20	11687025	*ADGRG1*	ENSCJAG00000011920	1.99E-06	0.01713
22	988671	*EFNA2*	ENSCJAG00000005546	2.06E-06	0.01732
20	27662976	*VAC14*	ENSCJAG00000014847	2.52E-06	0.02015
16	5683907	*MetazoaSRP*	ENSCJAG00000037430	2.57E-06	0.02018
GL285864.1	261	NA	NA	2.59E-06	0.02018
16	71400669	*WDYHV1*	ENSCJAG00000000865	2.92E-06	0.02073
6	152377322	NA	ENSCJAG00000004382	3.04E-06	0.02137
1	141811277	*ANKS6*	ENSCJAG00000011142	3.47E-06	0.02304
GL286251.1	47558	NA	ENSCJAG00000031964	3.50E-06	0.02304
13	15635804	NA	ENSCJAG00000020226	3.67E-06	0.02346
1	195085254	*FBXO7*	ENSCJAG00000006238	3.67E-06	0.02346
1	189497727	*TPST2*	ENSCJAG00000008612	3.97E-06	0.02453
4	159937586	NA	ENSCJAG00000022894	4.11E-06	0.02462
3	183762034	*CRMP1*	ENSCJAG00000010648	4.28E-06	0.02462
18	2316759	*PRKAB2*	ENSCJAG00000002530	4.46E-06	0.02504
12	15186696	*TNFRSF17*	ENSCJAG00000016037	4.63E-06	0.02553
22	5416191	*HSD11B1L*	ENSCJAG00000016948	6.24E-06	0.03025
19	24318939	*SYT2*	ENSCJAG00000006030	6.36E-06	0.03025
3	183761811	*CRMP1*	ENSCJAG00000010648	6.40E-06	0.03025
7	152246015	*MAB21L3*	ENSCJAG00000006194	6.51E-06	0.03025
22	1104834	*DAZAP1*	ENSCJAG00000005609	6.55E-06	0.03025
15	5271984	*KLHL24*	ENSCJAG00000021216	6.58E-06	0.03025
20	44359746	*DEF8*	ENSCJAG00000010490	7.12E-06	0.03162
8	95080471	*PRKAG2*	ENSCJAG00000020422	7.99E-06	0.03317
22	21451357	NA	ENSCJAG00000032315	8.54E-06	0.03392
1	198828190	NA	ENSCJAG00000022385	8.72E-06	0.03442
10	125596501	*EML1*	ENSCJAG00000006842	8.82E-06	0.03449
3	67058583	*U6*	ENSCJAG00000027016	9.00E-06	0.03486
9	21381896	*NA*	ENSCJAG00000025904	9.46E-06	0.03577
12	84494722	*PGAM1*	ENSCJAG00000016575	9.97E-06	0.03705
5	126698006	*SEPT9*	ENSCJAG00000013203	1.08E-05	0.03968
5	114123373	*PTRF*	ENSCJAG00000000406	1.10E-05	0.03988
Y	1988813	*SCAPER*	ENSCJAG00000010868	1.10E-05	0.03988
10	132055046	NA	ENSCJAG00000000003	1.11E-05	0.03992
16	91386366	*BAALC*	ENSCJAG00000016141	1.13E-05	0.04025
GL284703.1	79888	NA	NA	1.21E-05	0.04237
5	157776512	*TSC22D1*	ENSCJAG00000019808	1.24E-05	0.04319

1	177313674	NA	ENSCJAG00000017966	1.25E-05	0.04319
4	34401203	*BAK1*	ENSCJAG00000016047	1.25E-05	0.04319
ACFV01190020.1	1340	NA	NA	1.26E-05	0.04319
8	22818148	*U6*	ENSCJAG00000034393	1.39E-05	0.04601
2	57730252	*PDGFA*	ENSCJAG00000004948	1.40E-05	0.04601
17	61597034	*GADL1*	ENSCJAG00000004395	1.40E-05	0.04602
6	155718429	*HDAC4*	ENSCJAG00000009423	1.45E-05	0.04676
20	44133661	*VPS9D1*	ENSCJAG00000010650	1.50E-05	0.04799

Table S4

89 CpG sites significantly associated with maternal weight at gestational day 120 in descending order of P value with FDR <0.05.

Chromosome	Start Position	Gene Name	Gene ID	*P* value	FDR
1	207684919	NA	ENSCJAG00000018966	2.57E-10	0.00016
7	36083382	*TMEM240*	ENSCJAG00000037689	9.78E-10	0.00020
4	1639040	NA	ENSCJAG00000022438	1.23E-09	0.00020
1	177324707	NA	ENSCJAG00000017966	7.87E-09	0.00075
10	16507933	NA	ENSCJAG00000032743	2.84E-08	0.00177
2	3048585	*WBSCR17*	ENSCJAG00000005178	4.57E-08	0.00202
1	177956403	*VAV2*	ENSCJAG00000010024	4.74E-08	0.00202
12	118225906	NA	ENSCJAG00000014400	6.52E-08	0.00251
22	12418791	*CACNA1A*	ENSCJAG00000004458	8.45E-08	0.00287
6	155718341	*HDAC4*	ENSCJAG00000009423	8.63E-08	0.00287
5	140947022	*URAD*	ENSCJAG00000019404	1.23E-07	0.00310
GL284914.1	52816	NA	NA	1.32E-07	0.00312
6	155933814	NA	ENSCJAG00000034894	1.76E-07	0.00375
9	13850661	*CACNA1C*	ENSCJAG00000000732	2.63E-07	0.00479
12	108358469	*PPAPDC1A*	ENSCJAG00000006402	2.70E-07	0.00479
16	5683907	*MetazoaSRP*	ENSCJAG00000037430	2.78E-07	0.00481
4	34401203	*BAK1*	ENSCJAG00000016047	3.34E-07	0.00548
5	135692805	*5S_rRNA*	ENSCJAG00000024555	3.43E-07	0.00548
22	48099867	*ZNF211*	ENSCJAG00000000037	3.78E-07	0.00553
2	192181103	*CTNND2*	ENSCJAG00000009238	3.80E-07	0.00553
1	181715957	*EXD3*	ENSCJAG00000013170	4.03E-07	0.00572
13	10797937	*EPHX2*	ENSCJAG00000019677	5.40E-07	0.00720
7	49305869	*KAZN*	ENSCJAG00000008920	7.94E-07	0.00976
2	3048584	*WBSCR17*	ENSCJAG00000005178	8.34E-07	0.01005
3	186729794	NA	ENSCJAG00000031790	8.97E-07	0.01031
12	404895	*DECR2*	ENSCJAG00000011883	9.20E-07	0.01031
21	49728719	*COL6A2*	ENSCJAG00000001863	1.06E-06	0.01133
18	36565866	*SCYL3*	ENSCJAG00000010050	1.16E-06	0.01200

20	27662976	*VAC14*	ENSCJAG00000014847	1.25E-06	0.01223
19	24318939	*SYT2*	ENSCJAG00000006030	1.28E-06	0.01223
1	177313750	NA	ENSCJAG00000017966	1.37E-06	0.01289
1	177313680	NA	ENSCJAG00000017966	1.48E-06	0.01347
4	133634568	*EYA4*	ENSCJAG00000002817	1.97E-06	0.01713
1	177313674	NA	ENSCJAG00000017966	2.01E-06	0.01713
6	152377322	NA	ENSCJAG00000004382	2.40E-06	0.01941
22	988671	*EFNA2*	ENSCJAG00000005546	2.68E-06	0.02047
20	44359746	*DEF8*	ENSCJAG00000010490	2.70E-06	0.02047
11	130031608	*RNH1*	ENSCJAG00000012214	2.78E-06	0.02047
22	47093771	*ZNF471*	ENSCJAG00000020296	2.80E-06	0.02047
22	48494355	NA	ENSCJAG00000000381	3.16E-06	0.02169
1	195085254	*FBXO7*	ENSCJAG00000006238	3.37E-06	0.02290
4	159937586	NA	ENSCJAG00000022894	3.73E-06	0.02363
1	184797700	NA	ENSCJAG00000004879	3.93E-06	0.02453
15	5271984	*KLHL24*	ENSCJAG00000021216	3.99E-06	0.02453
5	135692806	*5S_rRNA*	ENSCJAG00000024555	4.15E-06	0.02462
1	141811277	*ANKS6*	ENSCJAG00000011142	4.23E-06	0.02462
13	15635804	NA	ENSCJAG00000020226	4.26E-06	0.02462
19	43294549	*7SK*	ENSCJAG00000032509	4.30E-06	0.02462
11	55289953	*FAM181B*	ENSCJAG00000014709	4.31E-06	0.02462
3	152412117	*U6*	ENSCJAG00000025583	4.48E-06	0.02504
16	71400669	*WDYHV1*	ENSCJAG00000000865	4.72E-06	0.02577
11	125324021	*FGF3*	ENSCJAG00000009730	5.79E-06	0.03025
4	163744391	NA	ENSCJAG00000012365	5.98E-06	0.03025
4	106452640	NA	ENSCJAG00000006178	6.05E-06	0.03025
1	177965978	*VAV2*	ENSCJAG00000010024	6.14E-06	0.03025
20	44133661	*VPS9D1*	ENSCJAG00000010650	6.17E-06	0.03025
16	12801023	*CA8*	ENSCJAG00000008976	6.26E-06	0.03025
22	21451357	NA	ENSCJAG00000032315	6.42E-06	0.03025
12	40118839	NA	ENSCJAG00000015397	6.43E-06	0.03025
15	65610998	*FBLN2*	ENSCJAG00000016873	6.48E-06	0.03025
5	114123373	*PTRF*	ENSCJAG00000000406	6.57E-06	0.03025
6	157494660	*CROCC2*	ENSCJAG00000006365	6.73E-06	0.03061
GL284914.1	50043	NA	NA	6.75E-06	0.03061
4	19855367	*SLC35B3*	ENSCJAG00000021320	7.06E-06	0.03155
5	157155159	NA	ENSCJAG00000017549	7.30E-06	0.03182
1	189497727	*TPST2*	ENSCJAG00000008612	7.40E-06	0.03182
7	54370823	*VWA5B1*	ENSCJAG00000006551	7.41E-06	0.03182
Y	1988813	*SCAPER*	ENSCJAG00000010868	7.49E-06	0.03182
GL285864.1	261	NA	NA	7.51E-06	0.03182
22	30047764	*ZNF146*	ENSCJAG00000009524	7.57E-06	0.03182
1	177788729	*SARDH*	ENSCJAG00000009864	7.57E-06	0.03182
22	5416191	*HSD11B1L*	ENSCJAG00000016948	7.78E-06	0.03249
ACFV01192733.1	1291	NA	NA	8.18E-06	0.03329
6	155718429	*HDAC4*	ENSCJAG00000009423	8.23E-06	0.03329

ACFV01192733.1	1207	NA	NA	8.85E-06	0.03449
4	30674268	NA	ENSCJAG00000020676	9.13E-06	0.03515
GL284900.1	12752	NA	NA	9.22E-06	0.03527
GL286232.1	66374	NA	ENSCJAG00000003723	9.95E-06	0.03705
6	46430125	*DLX2*	ENSCJAG00000007389	1.04E-05	0.03834
1	177385764	*SURF6*	ENSCJAG00000017951	1.16E-05	0.04109
12	15186696	*TNFRSF17*	ENSCJAG00000016037	1.27E-05	0.04327
11	113515486	*CLP1*	ENSCJAG00000008748	1.27E-05	0.04327
4	169752716	*NA*	ENSCJAG00000003511	1.29E-05	0.04366
11	123170411	*GRK2*	ENSCJAG00000001006	1.38E-05	0.04601
2	188068188	*FBXL7*	ENSCJAG00000010823	1.38E-05	0.04601
1	76580306	*BARX1*	ENSCJAG00000017399	1.42E-05	0.04634
6	158394771	*PDCD1*	ENSCJAG00000019795	1.44E-05	0.04668
ACFV01192733.1	1135	NA	NA	1.47E-05	0.04735
5	30031004	NA	ENSCJAG00000032266	1.52E-05	0.04799

Table S5

74 gene names that were significantly associated with maternal weight at any time point (p<0.05, FDR<0.05) along with their annotated molecular function and biological process obtained from DAVID (Huang, Sherman, & Lempicki, 2009a, 2009b)

Gene Name	Molecular Function	Biological Process
U6	No annotation (NA)	NA
Y_RNA	NA	NA
7SK	NA	NA
snoU13	NA	NA
5S_rRNA	NA	NA
Metazoa_SRP	NA	NA
BARX1	Sequence-specific DNA binding	Regulation of transcription, cell-cell signaling, negative regulation of WNT
ANKS6	NA	NA
SURF6	DNA binding, poly(A) RNA binding	ribosome biogenesis
SARDH	oxidoreductase activity	NA
VAV2	Rho guanyl-nucleotide exchange factor activity, metal ion binding	small GTPase mediated signal transduction, regulation of cell size, regulation of gene expression, cell migration, lamellipodium assembly, regulation of blood coagulation, regulation of Rho protein signal transduction, positive regulation of phosphatidylinositol 3-kinase activity

EXD3	nucleic acid binding, 3'-5' exonuclease activity	NA
TPST2	protein-tyrosine sulfotransferase activity	peptidyl-tyrosine sulfation
FBXO7	NA	mitophagy, protein targeting to mitochondrion, protein ubiquitination, regulation of protein stability, negative regulation of lymphocyte differentiation, negative regulation of cyclin-dependent protein serine/threonine kinase activity, negative regulation of G1/S transition of mitotic cell cycle
EML1	NA	microtubule cytoskeleton organization, hematopoietic progenitor cell differentiation, mitotic spindle organization, neuroblast proliferation, brain development
FAM181B	NA	NA
DGKZ	diacylglycerol kinase activity, ATP binding	protein kinase C-activating G-protein coupled receptor signaling pathway, intracellular signal transduction
CLP1	ATP binding, kinase activity, ATP-dependent polydeoxyribonucleotide 5'-hydroxyl-kinase activity, ATP-dependent polyribonucleotide 5'-hydroxyl-kinase activity	tRNA splicing, via endonucleolytic cleavage and ligation, cerebellar cortex development, targeting of mRNA for destruction involved in RNA interference, mRNA 3'-end processing, siRNA loading onto RISC involved in RNA interference
GRK2	G-protein coupled receptor kinase activity, ATP binding,	DNA damage checkpoint, desensitization of G-protein coupled receptor protein signaling pathway, negative regulation of the force of heart contraction by chemical signal, DNA repair, signal transduction, tachykinin receptor signaling pathway, heart development, peptidyl-serine phosphorylation, peptidyl-threonine phosphorylation, viral genome replication, receptor internalization, negative regulation of striated muscle contraction, viral entry into host cell, cardiac muscle contraction
FGF3		positive regulation of cell proliferation, fibroblast growth factor

		receptor signaling pathway, negative regulation of cardiac muscle tissue development
RNH1	ribonuclease inhibitor activity	regulation of angiogenesis
DECR2	Nucleoside disphosphate kinase activity, ATP binding; NADPH activity	GTP, UTP and CTP biosynthetic process, unsaturated fatty acid biosynthetic process
TNFRSF17	receptor activity,	lymphocyte homeostasis, signal transduction
PGAM1	bisphosphoglycerate mutase activity, bisphosphoglycerate 2-phosphatase activity, phosphoglycerate mutase activity	glycolytic process
PPAPDC1A	NA	NA
EPHX2	magnesium ion binding, catalytic activity, epoxide hydrolase activity, toxic substance binding, hydrolase activity, lipid phosphatase activity,	metabolic process, positive regulation of gene expression, cholesterol homeostasis, stilbene catabolic process, phospholipid dephosphorylation, regulation of cholesterol metabolic process
KLHL24	NA	regulation of kainate selective glutamate receptor activity
FBLN2	calcium ion binding, extracellular matrix binding	positive regulation of cell-substrate adhesion
CA8	carbonate dehydratase activity, zinc ion binding	one-carbon metabolic process, phosphatidylinositol-mediated signaling
WDYHV1	protein-N-terminal glutamine amidohydrolase activity	cellular protein modification process,
BAALC	NA	NA
GADL1	carboxy-lyase activity, pyridoxal phosphate binding	carboxylic acid metabolic process
PRKAB2	AMP-activated protein kinase activity	signal transduction, regulation of protein kinase activity
SCYL3	protein kinase activity, ATP binding	
SYT2	calcium-dependent phospholipid binding	neurotransmitter secretion, positive regulation of dendrite extension
WBSCR17	transferase activity, transferring glycosyl groups, carbohydrate binding	protein glycosylation
PDGFA		response to wounding, negative regulation of phosphatidylinositol biosynthetic process, negative regulation of platelet activation,

		positive regulation of phosphatidylinositol 3-kinase signaling, regulation of smooth muscle cell migration, positive regulation of protein autophosphorylation, positive regulation of metanephric mesenchymal cell migration by platelet-derived growth factor receptor-beta signaling pathway, positive regulation of MAP kinase activity, positive regulation of DNA replication, positive regulation of fibroblast proliferation, negative chemotaxis, positive regulation of protein kinase B signaling, positive regulation of ERK1 and ERK2 cascade
FBXL7		G2/M transition of mitotic cell cycle, mitotic nuclear division, cell proliferation, protein ubiquitination, SCF-dependent proteasomal ubiquitin-dependent protein catabolic process
CTNND2		single organismal cell-cell adhesion, synapse organization, regulation of canonical Wnt signaling pathway, dendritic spine morphogenesis
ADGRG1	G-protein coupled receptor activity, heparin binding, extracellular matrix binding	angiogenesis, cell adhesion, cell surface receptor signaling pathway, Rho protein signal transduction, negative regulation of cell proliferation, vascular endothelial growth factor production, cerebral cortex regionalization, layer formation in cerebral cortex, positive regulation of Rho protein signal transduction, positive regulation of cell adhesion, protein kinase C signaling, seminiferous tubule development, positive regulation of neural precursor cell proliferation, negative regulation of neuron migration
VAC14	NA	regulation of lipid kinase activity
VPS9D1	NA	NA

DEF8	metal ion binding	intracellular signal transduction
COL6A2		protein heterotrimerization
EFNA2	NA	osteoclast differentiation, bone remodeling, ephrin receptor signaling pathway
DAZAP1	Nucleotide binding, nucleic acid binding, RNA stem-loop binding, poly(A) RNA binding	Maternal placenta development, spermatogenesis, cell proliferation, positive regulation of mRNA splicing
HSD11B1L	oxidoreductase activity	NA
TRAPPC5	NA	Golgi vesicle transport
CACNA1A	high voltage-gated calcium channel activity	sulfur amino acid metabolic process, glucose metabolic process, positive regulation of cytosolic calcium ion concentration, gamma-aminobutyric acid signaling pathway, neuromuscular synaptic transmission, synapse assembly, adult walking behavior, cell death, gamma-aminobutyric acid secretion, regulation of acetylcholine secretion, neurotransmission, cell growth, regulation of calcium ion-dependent exocytosis, transmission of nerve impulse, spinal cord motor neuron differentiation, cerebellum maturation, cerebellar molecular layer development, cerebellar Purkinje cell differentiation, vestibular nucleus development, cellular chloride ion homeostasis, negative regulation of hormone biosynthetic process, synaptic transmission, glutamatergic, neurotransmitter metabolic process, hormone metabolic process, receptor clustering, negative regulation of neuron apoptotic process, behavioral response to pain, calcium ion-regulated exocytosis of neurotransmitter, dendrite morphogenesis, regulation of axonogenesis, musculoskeletal movement, spinal reflex action, neuromuscular process controlling balance, membrane depolarization, rhythmic synaptic transmission

ZNF146	nucleic acid binding, metal ion binding	NA
ZNF471	nucleic acid binding, metal ion binding	regulation of transcription, DNA-templated
ZNF211	NA	NA
CRMP1	hydrolase activity, acting on carbon-nitrogen (but not peptide) bonds	microtubule cytoskeleton organization, axon guidance, negative regulation of neuron projection development, negative regulation of actin filament binding
SLC35B3	NA	positive regulation of defense response to virus by host, transmembrane transport, mitophagy in response to mitochondrial depolarization, xenophagy
BAK1	NA	B cell homeostasis, protein processing in endoplasmic reticulum, limb morphogenesis, post-embryonic eye morphogenesis, vagina development, apoptotic processes, blood vessel apoptotic process
EYA4	Protein tyrosine phosphatase activity, metal ion binding	Regulation of transcription, multicellular oranism development, sensory perception of sound
PTRF	rRNA primary transcript binding, poly(A) RNA binding	transcription initiation from RNA polymerase I promoter, termination of RNA polymerase I transcription
SEPT9	GTP binding	cell cycle, protein heterooligomerization, positive regulation of nonmotile primary cilium assembly
URAD	carboxy-lyase activity	allantoin biosynthetic process
TSC22D1	transcription factor activity, sequence-specific DNA binding	NA
MCTP2	calcium ion binding	NA
DLX2	RNA polymerase II regulatory region sequence-specific DNA binding, transcriptional activator activity, RNA polymerase II transcription regulatory region sequence-specific binding, chromatin binding, single-stranded RNA	negative regulation of transcription from RNA polymerase II promoter, regulation of transcription, DNA-templated, proximal/distal pattern formation, subpallium development, hippocampus development, olfactory bulb development, regulation of transcription from RNA polymerase

	binding, sequence-specific DNA binding	II promoter involved in forebrain neuron fate commitment, cerebral cortex GABAergic interneuron fate commitment, odontogenesis of dentin-containing tooth, negative regulation of Notch signaling pathway, embryonic cranial skeleton morphogenesis, negative regulation of oligodendrocyte differentiation, branching morphogenesis of a nerve, cartilage development
HDAC4	core promoter binding, chromatin binding, transcription corepressor activity, histone deacetylase activity, zinc ion binding, potassium ion binding, NAD-dependent histone deacetylase activity (H3-K14 specific), sequence-specific DNA binding, metal ion binding	negative regulation of transcription from RNA polymerase II promoter, skeletal system development, osteoblast development, chromatin remodeling, transcription, DNA-templated, regulation of transcription, DNA-templated, positive regulation of cell proliferation, negative regulation of cell proliferation, negative regulation of myotube differentiation, regulation of cardiac muscle contraction by calcium ion signaling, response to denervation involved in regulation of muscle adaptation, positive regulation of protein sumoylation, peptidyl-lysine deacetylation, regulation of gene expression, epigenetic, regulation of protein binding, negative regulation of sequence-specific DNA binding transcription factor activity, negative regulation of osteoblast differentiation, negative regulation of glycolytic process, positive regulation of transcription from RNA polymerase II promoter, regulation of skeletal muscle fiber development, positive regulation of sequence-specific DNA binding transcription factor activity, response to interleukin-1, histone H4 deacetylation
CROCC2	NA	NA

PDCD1		negative regulation of tolerance induction, negative regulation of apoptotic process, positive regulation of T cell apoptotic process
TMEM240	NA	NA
TNFRSF14	Receptor activity	positive regulation of cytokine secretion involved in immune response, negative regulation of alpha-beta T cell proliferation, positive regulation of peptidyl-tyrosine phosphorylation, defense response to Gram-negative bacterium, defense response to Gram-positive bacterium, positive regulation of T cell migration
KAZN	NA	NA
VWA5B1	NA	NA
MAB21L3	NA	NA
PRKAG2	cAMP-dependent protein kinase inhibitor activity, ATP binding, cAMP-dependent protein kinase regulator activity, phosphorylase kinase regulator activity, kinase activity, protein kinase activator activity, ADP binding	glycogen metabolic process, regulation of glycolytic process, positive regulation of peptidyl-threonine phosphorylation, regulation of fatty acid metabolic process, intracellular signal transduction
CACNA1C	voltage-gated calcium channel activity, high voltage-gated calcium channel activity, voltage-gated calcium channel activity involved in AV node cell action potential	immune system development, positive regulation of cytosolic calcium ion concentration, heart development, embryonic forelimb morphogenesis, camera-type eye development, calcium ion transmembrane transport via high voltage-gated calcium channel, regulation of heart rate by cardiac conduction, regulation of ventricular cardiac muscle cell action potential, membrane depolarization during atrial cardiac muscle cell action potential
ANKS1B	NA	regulation of synaptic plasticity by receptor localization to synapse
SCAPER	nucleic acid binding, zinc ion binding	NA
